# Bivalent separation into univalents precedes age-related meiosis I errors in oocytes

**DOI:** 10.1038/ncomms8550

**Published:** 2015-07-01

**Authors:** Yogo Sakakibara, Shu Hashimoto, Yoshiharu Nakaoka, Anna Kouznetsova, Christer Höög, Tomoya S. Kitajima

**Affiliations:** 1 Laboratory for Chromosome Segregation, RIKEN Center for Developmental Biology, Kobe 650-0047, Japan; 2 IVF Namba Clinic, Osaka 550-0015, Japan; 3 Department of Cell and Molecular Biology, Karolinska Institutet, Stockholm 171 77, Sweden

## Abstract

The frequency of chromosome segregation errors during meiosis I (MI) in oocytes increases with age. The two-hit model suggests that errors are caused by the combination of a first hit that creates susceptible crossover configurations and a second hit comprising an age-related reduction in chromosome cohesion. This model predicts an age-related increase in univalents, but direct evidence of this phenomenon as a major cause of segregation errors has been lacking. Here, we provide the first live analysis of single chromosomes undergoing segregation errors during MI in the oocytes of naturally aged mice. Chromosome tracking reveals that 80% of the errors are preceded by bivalent separation into univalents. The set of the univalents is biased towards balanced and unbalanced predivision of sister chromatids during MI. Moreover, we find univalents predisposed to predivision in human oocytes. This study defines premature bivalent separation into univalents as the primary defect responsible for age-related aneuploidy.

## Abstract (web summary)

As oocytes age the frequency of chromosome segregation errors during meiosis I increases. Here the authors use live imaging of oocytes from naturally aged mice to provide direct evidence that bivalent separation into univalents is the primary defect responsible for age-related aneuploidy.

## Graphical abstract

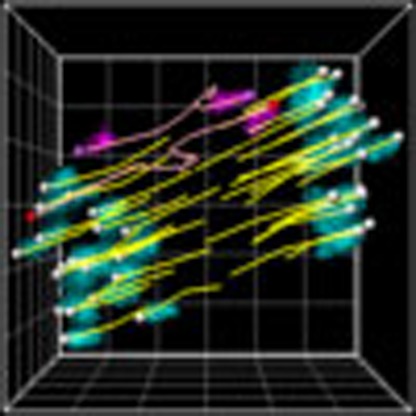

Aneuploidy is a leading cause of miscarriage and congenital diseases such as Down syndrome. Most aneuploid chromosomes originate from segregation errors during meiosis I (MI) in oocytes, and the frequency of the errors increases with age^1^,^2^,^3^,^4^. The two-hit model suggests that segregation errors during MI are caused by the combination of a first hit that creates susceptible crossover configurations during the foetal stage and a second hit comprising an age-related reduction in chromosome cohesion during the dictyate stage^1^,^5^,^6^,^7^,^8^. One prediction of this model is the existence of an age-related increase in univalents. Consistent with this prediction, *Smc1b* knockout oocytes, which have a reduced amount of cohesin, exhibit an age-related increase in univalents^9^. However, direct evidence of univalents as a major cause of segregation errors in the oocytes of naturally aged animals has been lacking^10^,^11^,^12^,^13^,^14^,^15^. Although an age-related increase in univalents in the oocytes of naturally aged mice was first suggested over four decades ago^16^, it was argued that most, if not all, of the univalents observed in that study were artefacts of chromosome spread^10^,^11^,^12^. Recent studies found univalents in a very small fraction of the oocytes of naturally aged mice^13^,^14^, which seemed unlikely to account for the majority of chromosome segregation errors during MI. These and other studies found weakly associated bivalents in a significant fraction of the oocytes of naturally aged mice^13^,^14^,^15^, but these bivalents undergo normal segregation during MI^13^. Instead, available evidence from naturally aged mice led to the proposal that an age-related reduction in cohesion between sister kinetochores (KTs)^14^,^15^ and defects in KT–microtubule interaction^17^ are leading causes of segregation errors during MI. Thus, the actual cause of segregation errors during MI in the oocytes of naturally aged animals remains unclear.

Here, we provide direct evidence that premature bivalent separation into univalents is the major cause of chromosome segregation errors during MI. By using high-resolution and high-throughput live imaging microscopy and complete three-dimensional (3D) tracking of KTs in the oocytes of naturally aged mice, we show that the vast majority of chromosome segregation errors are preceded by bivalent hyperstretching and subsequent separation into univalents. The univalents are strongly biased towards predivision of sister chromatids. We provide direct observation of balanced predivision of sister chromatids during MI, which was hypothesized two decades ago^5^, as a major type of age-related chromosome segregation errors. Consistent with observations in mouse oocytes, human oocytes exhibit univalents that are predisposed to predivision of sister chromatids during MI.

## Results

### Direct observation of age-related MI errors

**Figure 1:**
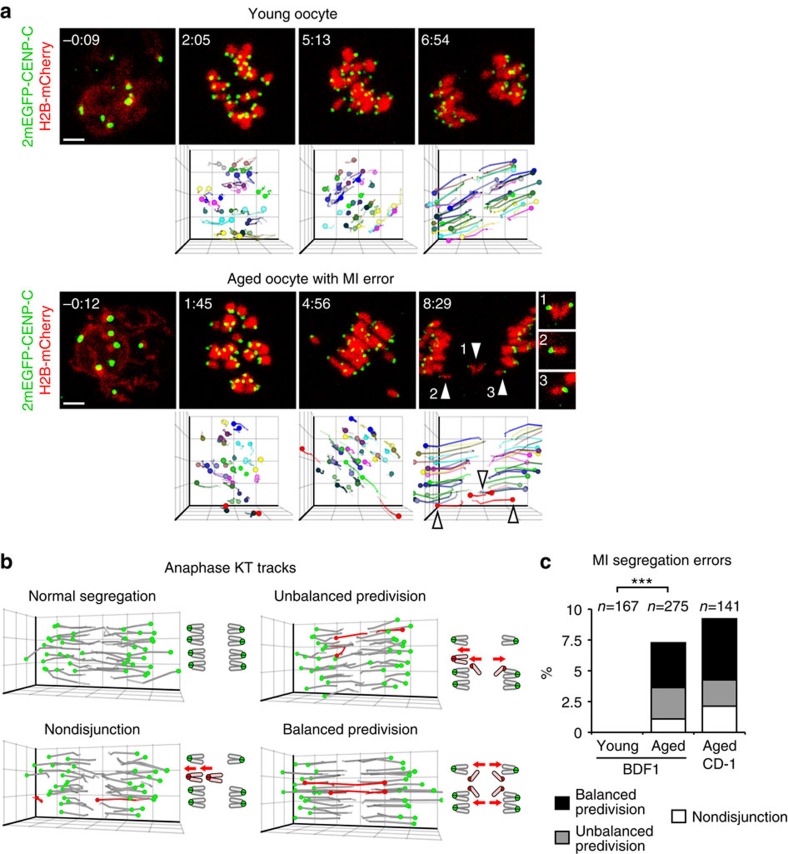
Live imaging of MI errors in aged mouse oocytes. (**a**) Live imaging and tracking of KTs. Maximum *z*-projection images of 2mEGFP-CENP-C (KTs, green) and H2B-mCherry (chromosomes, red) in the oocytes of young (2 months old) and aged (16 months old) BDF1 mice. KT signals are peak-enhanced and background-subtracted. The 3D plots show KT positions (spheres) and their tracks (lines). Same colours were used for homologous KTs. The aged oocyte underwent balanced predivision of sister chromatids (arrowheads and insets). Time after NEBD (h:mm). Scale bar, 5 μm. See also Supplementary Movie 2. (**b**) Distinct types of segregation errors during MI. Red spheres and lines indicate KTs that have segregated abnormally. See also Supplementary Fig. 1. (**c**) Balanced and unbalanced predivision are predominant over nondisjunction. The rates of segregation errors during MI in the oocytes from young BDF1, aged BDF1, and aged CD-1 (11 months old) mice. Fisher's exact test was performed. ***P<0.001.

There are potentially three distinct types of segregation errors during MI: nondisjunction of homologous chromosomes (4:0 segregation), unbalanced predivision of sister chromatids (3:1 segregation) and balanced predivision of sister chromatids (2:2 segregation) (Supplementary Fig. 1). Balanced predivision^5^, in which both homologous chromosomes undergo sister chromatid segregation during MI, has not yet been directly demonstrated because it is indistinguishable from premature sister chromatid separation during meiosis II (MII) by conventional approaches using fixed metaphase II oocytes. To directly observe chromosome segregation errors, we established a high-resolution and high-throughput imaging of KTs and chromosomes in live mouse oocytes (Fig. 1a and Supplementary Movie 1)^18^. We generated four-dimensional data sets throughout MI for 275 ‘aged' oocytes obtained from 16-month-old BDF1 mice and for 167 ‘young' oocytes obtained from 2-month-old mice (Supplementary Table 1). The data sets allowed us to robustly track all KTs in 3D from prometaphase to anaphase I (Fig. 1a, Supplementary Movie 2)^18^. Manual assessment of all KT trajectories during anaphase I (Fig. 1b) indicated that 20/275 aged oocytes (7.3%) underwent segregation errors during MI, whereas no young oocytes did (0/167; Fig. 1c and Supplementary Table 1). This KT tracking analysis revealed that balanced predivision of sister chromatids (9/20, 45%) and unbalanced predivision of sister chromatids (7/20, 35%) are predominant over nondisjunction of homologous chromosomes (4/20, 20%) (Fig. 1c). Similar results were obtained for oocytes from aged CD-1 mice (7/13, 54% balanced predivision; 3/13, 23% unbalanced predivision; and 3/13, 23% nondisjunction) (Fig. 1c). Counting the number of KTs in aged BDF1 oocytes fixed at metaphase II suggested that live imaging had no adverse effect on the rates of unbalanced predivision and nondisjunction (Supplementary Fig. 2 and Supplementary Movie 3). These results indicate that segregation errors during MI are strongly biased towards predivision of sister chromatids, including balanced predivision.

### Bivalent separation into univalents precedes MI errors

**Figure 2:**
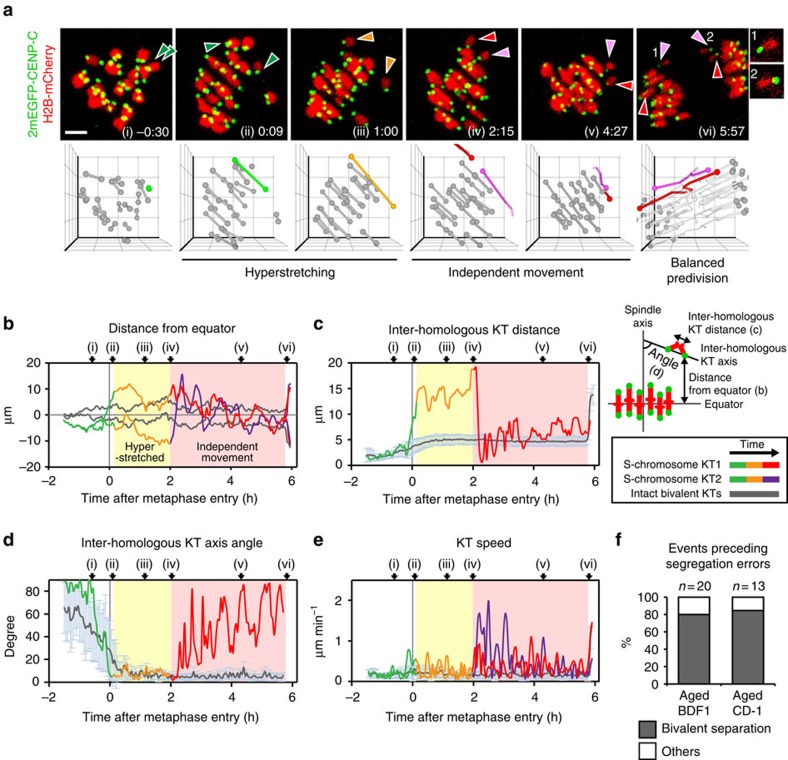
Bivalent separation prior to chromosome segregation errors. (**a**) Bivalent hyperstretching and separation before segregation errors. Maximum *z*-projection images of aged (16 months old) BDF1 oocytes expressing 2mEGFP-CENP-C (KTs, green) and H2B-mCherry (chromosomes, red). KT signals are peak-enhanced and background-subtracted. Arrowheads indicate S-chromosome KTs. The 3D plots show KT positions (spheres) and the connection between homologous KTs (lines). In (i)−(ii), the S-chromosome is coloured in green. In (iii), the hyperstretched S-chromosome is coloured in orange. In (iv)−(vi), the positions (spheres) and trajectories (lines) of the S-chromosome KTs exhibiting independent movements are coloured in red and purple. Intact bivalents are coloured in grey. The insets show the predivision of sister chromatids. Note that the spindle rotated before (vi). Time after metaphase entry (h:mm). Scale bar, 5 μm. See also Supplementary Movie 4. (**b–e**) Characteristic S-chromosome dynamics suggests bivalent hyperstretching followed by premature separation. KT distance from the spindle equator (**b**), inter-homologous KT distance (**c**), angle between inter-homologous KT axis and the spindle axis (**d**) and KT speed (**e**). The data for S-chromosomes are coloured as in **a**. The data for bivalents are coloured in grey. Error bars, s.d. (**f**) Bivalent separation precedes the majority of segregation errors during MI. The KT trajectories that underwent segregation errors in oocytes from aged BDF1 (16 months old) and CD-1 (11 months old) mice were analysed.

Strikingly, analysis of the KT trajectories of the chromosomes later associated with segregation errors suggested premature bivalent separation (Supplementary Movie 4). Hereafter, we refer to these chromosomes as ‘S-chromosomes'. Before metaphase I, an S-chromosome appeared as a normal bivalent with a pair of homologous KTs (Fig. 2a(i)). Upon entry into metaphase I, the S-chromosome bivalent stretched with a similar timing to that of the other bivalents (Fig. 2a–c(ii)). This stretching resulted in an extremely long distance between the homologous KTs (inter-homologous KT distance; Fig. 2a,c(iii)). Although no detectable H2B-mCherry signal was observed in the middle of the hyperstretched bivalent (Fig. 2a(iii)), it was possible that the homologous chromosomes were still functionally connected because the line connecting the homologous KTs (inter-homologous KT axis) was stably oriented along the spindle axis for a maximum of 233 min (Fig. 2b,d and Supplementary Fig. 3). This observation of hyperstretched bivalents is consistent with a previous report^13^. Thereafter, however, we found that the homologous KTs of the S-chromosome suddenly began to oscillate independently between the spindle poles at an increased speed (Fig. 2a–e(iv) and Supplementary Movie 4). The characteristic profiles of this oscillation were indistinguishable from those of univalents generated due to a defect in synaptonemal complex formation in the oocytes of young *Sycp3* knockout mice (Supplementary Fig. 4 and Supplementary Movie 5)^19^. We therefore functionally define these apparently separated S-chromosome units exhibiting independent oscillation as univalents. Importantly, of the 20 chromosomes that exhibited segregation errors during MI in aged BDF1 oocytes, 16 (80%) showed bivalent hyperstretching followed by separation into univalents (Fig. 2f, Supplementary Fig. 3 and Supplementary Table 1). The same phenomenon preceded 85% (11/13) of segregation errors during MI in oocytes from aged CD-1 mice (Fig. 2f and Supplementary Fig. 5). These results suggest that premature bivalent separation into univalents precedes the majority of chromosome segregation errors during MI in the oocytes of aged mice.

### Age-related increase in univalents in mouse oocytes

**Figure 3:**
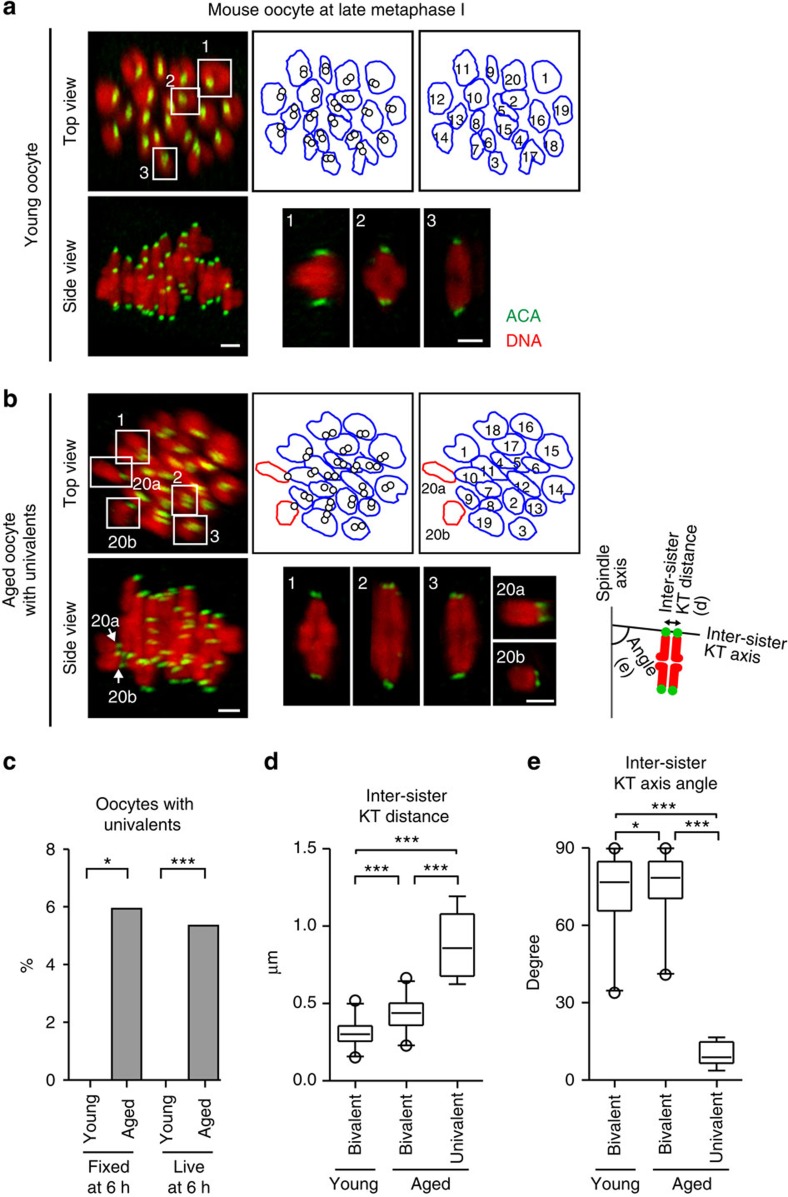
Age-related increase in univalents in mouse oocytes. (**a**,**b**) Univalents in fixed aged oocytes. Oocytes from young (2 months old (**a**)) and aged (16 months old (**b**)) BDF1 mice were cultured and fixed at 6 h after NEBD. The oocytes were stained for KTs (ACA, green) and DNA (Hoechst 33342, red). KT signals are peak-enhanced and background-subtracted. All homologous KT positions were determined in 3D and the number of chromosomes was counted. Projection views from the top and side of the spindle are shown with signal interpolation in *z*. The outlines of chromosomes (bivalents, blue; univalents, red) and the positions of homologous KTs (circles) are shown. Representative bivalents and univalents are magnified. Scale bars, 2 μm. See also Supplementary Movie 6. (**c**) The rate of oocytes with univalents. In fixed oocytes, separated chromosome units that have one of the homologous KTs and an inter-homologous KT axis with a tilt angle of >25° with respect to the spindle axis were categorized as univalents (*n*=106, 118). The same criteria was used for live oocytes at 6 h after NEBD (*n*=180, 289). Note that the rates of univalents presented here is underestimated because this criteria excludes univalents that are indistinguishable from hyperstretched bivalents at a single time point. The rate of univalents determined by an analysis of multiple time points in live aged oocytes was 9.5% (Supplementary Table 1). Fisher's exact test was performed. *P=0.015, ***P=0.0004. (**d,e**) Univalents are predisposed to predivision. The inter-sister KT distance (**c**) and the angle between inter-sister KT axis and the spindle axis (**d**) were measured (*n*=160, 152, 8). Boxes show the 25th–75th percentiles and whiskers show 1–99 percentiles. Two-tailed, unpaired Student's *t*-test was performed. *P<0.05, ***P<0.001.

To confirm the age-related increase in univalents, we fixed and immunostained oocytes at late metaphase I without chromosome spreading. Determination of all KT positions in 3D revealed that a significant fraction of aged oocytes had univalents, whereas no young oocytes did (Fig. 3a–c and Supplementary Movie 6). The rate of univalents remained consistent between fixed and live oocytes (Fig. 3c). The sister KTs of these univalents were frequently bioriented and aligned on the metaphase plate (Fig. 3b,d,e), which was consistent with the observation of univalents that later exhibited sister chromatid predivision in live aged oocytes (Supplementary Fig. 6). These results confirm the age-related increase in univalents as the major cause of segregation errors during MI in mouse oocytes.

### Univalents in human oocytes

**Figure 4:**
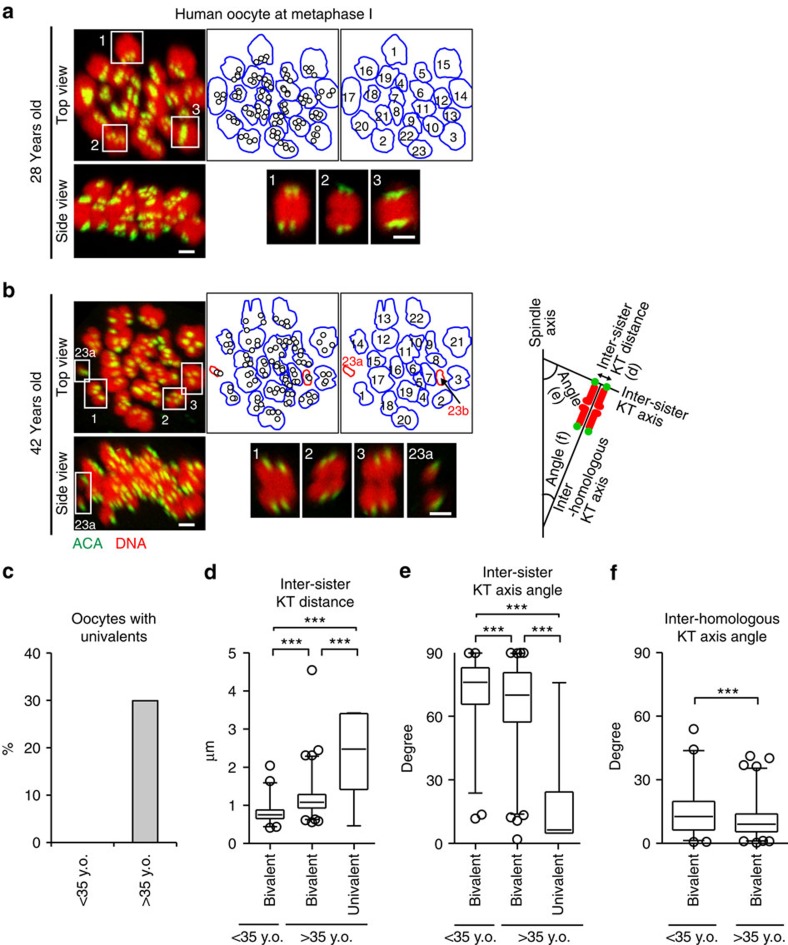
Univalents predisposed to predivision in human oocytes. (**a**,**b**) Univalents in human oocytes. Oocytes were stained for KTs (ACA, green) and DNA (Hoechst 33342, red). Oocytes from 28-year old (**a**) and 42-year-old (**b**) patients are shown. KT signals are peak-enhanced and background-subtracted. All sister and homologous KT positions were determined in 3D and the number of chromosomes was counted. Projection views from the top and side of the spindle are shown with signal interpolation in *z*. The outlines of chromosomes (bivalents, blue; univalents, red) and the positions of KTs (circles) are shown. Representative bivalents and univalents are magnified. Scale bars, 2 μm. See also Supplementary Movies 7 and 8. (**c**) The rate of oocytes with univalents. Separated chromosome units that have one of the homologous KTs and an inter-homologous KT axis with a tilt angle of >25° with respect to the spindle axis were categorized as univalents (*n*=6, 10). (**d**,**e**) Univalents are predisposed to predivision. The inter-sister KT distance (**d**) and the angle between the inter-sister KT axis and the spindle axis (**e**) were measured (*n*=276, 454, 6). (**f**) Homologous chromosome biorientation. The angle between the inter-homologous KT axis and the spindle axis was measured (*n*=276, 454). Boxes show the 25th–75th percentiles and whiskers show 1–99 percentiles. Two-tailed, unpaired Student's *t*-test was performed. ***P<0.001. y.o., years old.

To investigate whether univalents are observed in human oocytes, we fixed and immunostained oocytes at MI from patients of different ages without chromosome spreading. Determination of all KT positions in 3D (Fig. 4a and Supplementary Movie 7) revealed that among 16 oocytes carrying a metaphase plate, three oocytes from relatively old patients contained univalents (Fig. 4b,c and Supplementary Movie 8). The sister KTs of the univalents were frequently bioriented and aligned on the metaphase plate (Fig. 4b,d,e), which suggests a predisposition to sister chromatid predivision during MI. On bivalents, the inter-sister KT distance was significantly greater in oocytes from older patients (Fig. 4d), as observed in mouse oocytes (Fig. 3d)^14^,^15^. This age-related reduction in sister KT cohesion had a significant but relatively small impact on the capacity of bivalents to biorient the homologous KTs (Fig. 4f) and to monoorient the sister KTs (Fig. 4e), whereas univalent formation strongly biased towards biorientation of the sister KTs (Fig. 4e). These results suggest that univalent formation, presumably through premature bivalent separation as observed in mouse oocytes, is a critical event for chromosome segregation errors during MI in human oocytes.

## Discussion

It has long been speculated that an age-related increase in univalents might be a leading cause of chromosome segregation errors in oocytes^5^,^6^,^7^,^9^,^16^,^19^. In 1968, Henderson and Edwards^16^ observed an age-related increase in univalents in chromosome spread samples of the oocytes from naturally aged mice. Recent studies detected univalents in the oocytes from naturally aged mice without chromosome spreading^13^,^14^, although the sample size in these studies was insufficient to show statistical significance and the rate of the detected univalents did not match the rate of chromosome segregation errors. Instead, these and other studies observed a significant age-related increase in weakly associated bivalents and sister KTs^13^,^14^,^15^, raising the possibility that these defects are a leading cause of chromosome segregation errors.

**Figure 5:**
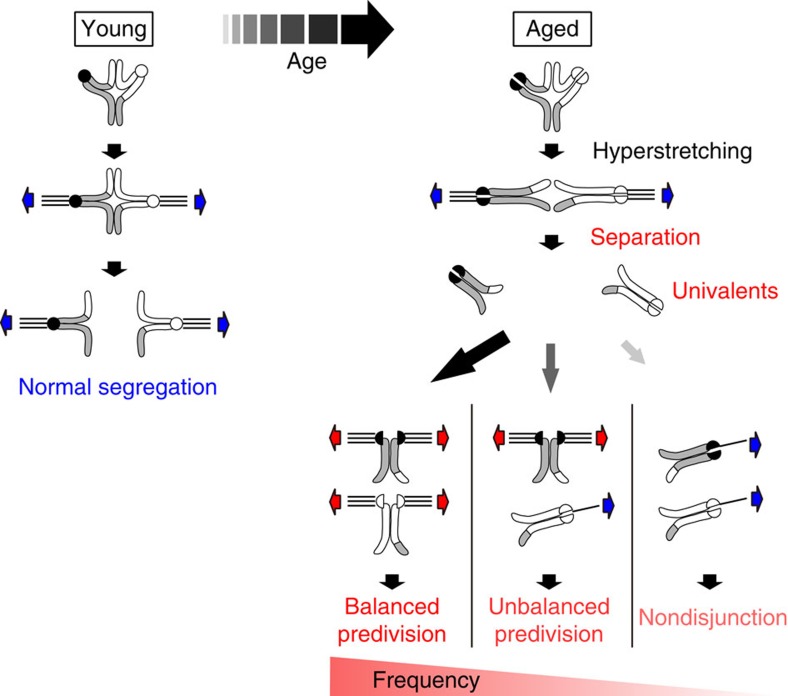
Premature bivalent separation into univalents precedes age-related chromosome segregation errors during MI. Bivalents with weakened cohesion in aged oocytes undergo hyperstretching and separation into univalents by microtubule-mediated bipolar spindle forces. This bipolar forces in turn biorient the sister KTs of the univalents. The univalents are therefore strongly biased towards predivision of sister chromatids, including balanced predivision. After balanced predivision during MI, the chromatids undergo random segregation during MII.

In the present study, our systematic analyses of the dynamics and structures of single chromosomes in the oocytes of naturally aged mice provided direct evidence that premature bivalent separation into univalents is the major cause of age-related chromosome segregation errors during MI (Fig. 5). Consistent with this finding, we observed univalents predisposed to segregation errors in human oocytes, although this observation should be interpreted cautiously because oocytes that remained immature after hormonal stimulation were used in this study. In mouse oocytes, a small fraction of segregation errors were not preceded by premature bivalent separation (4/20, 20% in BDF1; 2/13, 15% in CD-1; Supplementary Table 1). Future studies are needed to determine whether these errors are attributed to an age-related loss of sister KT cohesion on intact bivalents^14^,^15^ or other defects that facilitate incorrect KT–microtubule attachments^17^.

Our observation of premature bivalent separation into univalents is consistent with a prediction of the two-hit model^1^,^5^,^6^,^7^,^8^. Our findings suggest that the functional connection between the homologous chromosomes of bivalents subjected to both hits is resolved by microtubule-mediated forces for bivalent stretching, which thus results in production of univalents (Fig. 5). The possibility that a leak of separase activity contributed to the bivalent separation cannot be excluded. The sister KTs of the univalents are strongly biased towards bipolar microtubule attachment, possibly for two reasons. First, monopolar microtubule attachments to the sister KTs fail to be stabilized due to a loss of tension between homologous KTs, whereas bipolar attachments can be stabilized by the tension between sister KTs^19^,^20^. Second, an age-related loss of sister KT cohesion^14^,^15^ may disrupt MI-specific sister KT geometry that is known to be essential for univalents, but largely dispensable for bivalents, to make monopolar microtubule attachments to the sister KTs^21^,^22^.

Consequently, the set of the univalents frequently results in balanced and unbalanced predivision of sister chromatids during MI (Fig. 5). Unbalanced predivision is consistent with the aneuploidy observed in human metaphase II oocytes^5^,^7^,^23^,^24^,^25^,^26^,^27^. Furthermore, balanced predivision, which was demonstrated to be the most predominant type of segregation errors during MI in mouse oocytes in this study, may reconcile the available human data. DNA polymorphism analyses of human trisomies have shown that most aneuploid chromosomes are caused by segregation errors during MI^1^,^2^,^3^,^4^, whereas genome-based DNA analyses of human oocytes reveal that chromosome number abnormalities are more frequent after MII than after MI^25^,^28^. This apparent discrepancy can be reconciled by balanced predivision during MI, which results in the normal number of chromatids after MI but a predisposition to an abnormal number after MII. Taking these findings together, we suggest that premature bivalent separation into univalents is a predominant cause of age-related segregation errors during MI in human oocytes. This study provides a basis for the development of strategies to prevent age-related production of aneuploid eggs, such as the introduction of an artificial tie that could physically secure homologous chromosomes until the onset of anaphase I in maturing oocytes.

## Methods

### Oocyte culture and microinjection of mRNA

Two- or 16-month-old BDF1, 11-month-old CD-1 and 2-month-old *Sycp3*^*−/−*^ (in C57BL/6 background) female mice were primed with an injection of pregnant mare's serum gonadotropin (Serotoropin, ASKA Pharmaceutical). Ovaries were excised and oocytes at the germinal vesicle stage were collected. The collected oocytes were cultured in M2 medium supplemented with 200 nM 3-isobutyl-1-metyl-xanthine (IBMX, Sigma). *In vitro*-transcribed mRNAs (1.2 pl of 650 ng μl^−1^ 2mEGFP-CENP-C and 0.6 pl of 350 ng μl^−1^ H2B-mCherry) were microinjected into oocytes. The microinjected oocytes were cultured for 2.5−3.0 h and then released to undergo meiotic maturation through the removal of IBMX from the culture. Oocytes that expressed a moderate level of H2B-mCherry at the germinal vesicle stage were used for further analysis. All experiments involving mice were approved (AH26-04) and carried out under the guidelines of RIKEN CDB.

### Time-lapse imaging data acquisition

Time-lapse imaging was performed as previously described^18^ with some modifications. Briefly, a Zeiss LSM710 confocal microscope equipped with a 40 × C-Apochromat 1.2NA water immersion objective lens (Carl Zeiss) was controlled by a multi-position autofocus macro^29^, which allowed us to record a maximum 32 oocytes in one experiment. Seventeen confocal *z*-sections (every 1.5 μm) of 256 × 256 pixel *xy* images covering a total volume of 30.4 × 30.4 × 25.5 μm were acquired at 3-min intervals for at least 12 h after inducing a resumption of meiosis. The KT signals were peak-enhanced and background-subtracted as previously described^18^.

### KT tracking

KT tracking was performed as previously described^18^. The distance between homologous KTs (inter-homologous KT distance) at 15 min before the onset of anaphase I was defined as the final inter-homologous KT distance. The inter-homologous KT distance at each time point was normalized to the final inter-homologous KT distance. Metaphase entry was defined as the time when >50% of bivalents maintained a minimum inter-homologous KT distance that exceeded 70% for three consecutive time points. Once the inter-homologous KT distance of a bivalent has exceeded 12 μm, this bivalent was defined as hyperstretched. Once the homologous KTs of a bivalent initiated independent oscillation, this bivalent was defined as separated into univalents.

The centre of the spindle was defined as the centre of mass of all KT positions. The orientation of the spindle axis was defined as the average orientation of the inter-homologous KT axes of bivalents showing an inter-homologous KT distance >70%. The KT positions of univalents were eliminated from the calculations of the centre and axis of the spindle. The plane orthogonal to the spindle axis and passing through the spindle centre was defined as the spindle equator. The spindle equator at the time of metaphase entry was used for all time points before the metaphase entry.

To measure the oscillation amplitude, oscillation frequency and KT speed, the KT tracks were fitted with a cubic smoothing spline. The initiation of independent movements of separated chromosome units was defined as the point at which the KT began to exhibit consecutive anti-poleward movement over three time points, followed by a displacement of over 10 μm within 15 min. The time point when a KT began to exhibit consecutive poleward or anti-poleward movement over three time points was defined as the turning point. The oscillation amplitude was defined as the KT displacement along the spindle axis from a turning point to the next. The oscillation frequency was defined as the number of turning points after a KT displacement over 10 μm.

### Immunostaining of mouse oocytes

Oocytes that underwent nuclear envelope breakdown (NEBD) within 60 min after the induction of meiotic resumption were collected either 6 h after NEBD (late metaphase I) or 12–14 h after NEBD (metaphase II). The oocytes were fixed with 1.6% paraformaldehyde in 100 mM PIPES pH 7.0, 1 mM MgCl_2_ and 0.1% Triton X-100 for 30 min at room temperature. The fixed oocytes were washed three times with PBT (0.1% Triton X-100 in PBS) and then blocked with 3% BSA–PBT at 4 °C overnight. The oocytes were then incubated with human anti-centromere antibodies (ACA, Antibodies Incorporated, 15-234) at 1:100 in 3% BSA–PBT at 4 °C overnight. The oocytes were subsequently washed three times with 3% BSA–PBT and incubated with an Alexa Fluor 488 goat anti-human antibody (Molecular Probes, A11013) at 1:500 in 3% BSA–PBT at room temperature for 4 h in the dark. DNA was counterstained with 40 μg ml^−1^ Hoechst 33342. The oocytes were finally washed again and transferred to 0.01% BSA–PBS for imaging with a Zeiss LSM780 confocal microscope. Confocal *z*-sections (every 0.2 μm) of 512 × 512 pixel *xy* images covering the total volume of the spindle were acquired. The images were reconstructed into 3D with Imaris software (Bitplane).

### Human oocyte experiments

Human oocyte experiments were approved by institutional human research ethics committees at RIKEN (KOBE-IRB-13-22) and IVF Namba Clinic (2014-1), registered in Japan Society of Obstetrics and Gynecology (registry number 132), and carried out under these guidelines. Immature oocytes that were not used for fertility treatment were donated after patients received a full explanation of the experiments and provided signed informed consent.

Controlled ovarian stimulation was performed by using standard protocols with modifications according to the patients' medical history. Oocytes were picked up by transvaginal aspiration with an 18 gauge single lumen needle (Smiths Medical) 36 h after human chorionic gonadotropin injection. All mature oocytes were inseminated by intracytoplasmic sperm injection for fertility treatment. Oocytes that were still immature at 42 h after human chorionic gonadotropin injection were donated for this study. The immature oocytes were fixed in 2% paraformaldehyde solution at 37 °C for 30 min. Immunostaining was performed by the same procedure that was used for mouse oocytes except for primary antibody incubation for two overnights. The oocytes were imaged with a Zeiss LSM780 confocal microscope equipped with a 63 × C-Apochromat 1.2NA water immersion objective lens. To remove oocytes that were degenerate or otherwise aberrant, we selected oocytes that exhibit a metaphase plate with or without misaligned chromosomes for high-resolution imaging. Confocal *z*-sections (every 0.2 μm) of 512 × 512 pixel *xy* images covering the total volume of the spindle were acquired. The images were reconstructed into 3D with Imaris software.

## Additional information

**How to cite this article:** Sakakibara, Y. *et al.* Bivalent separation into univalents precedes age-related meiosis I errors in oocytes. *Nat. Commun.* 6:7550 doi: 10.1038/ncomms8550 (2015).

## Supplementary Material

Supplementary Information Supplementary Figures 1-6 and Supplementary Tables 1.

Supplementary Movie 1 Live imaging of young BDF1 oocytes expressing 2mEGFP-CENP-C (green) and H2BmCherry (red). Images of 32 oocytes acquired in one experiment are shown. Time elapsed (hh:mm). Scale bar, 10 μm

Supplementary Movie 2 Images of an aged BDF1 oocyte expressing 2mEGFP-CENP-C (green) and H2BmCherry (blue) were reconstructed into 3D. All KTs were detected and tracked throughout MI. Colored spots indicate the KTs of a chromosome that underwent balanced predivision. Grid unit, 5 μm. Time after NEBD (hh:mm). See also Fig. 1a.

Supplementary Movie 3 Images of an aged BDF1 oocyte immunostained for KTs (ACA, green) and DNA (Hoechst33342, red) at metaphase II were reconstructed into 3D. This oocyte had 41 KTs. Grid unit, 2 μm. See also Supplementary Fig. 2a.

Supplementary Movie 4 Images of a BDF1 aged oocyte expressing 2mEGFP-CENP-C (green) and H2BmCherry (blue) were reconstructed into 3D. All homologous KTs were detected and tracked throughout MI. KT positions (spheres) and the connection of homologous KTs (lines). The S-chromosome is colored in green. The hyperstretched S-chromosome is colored in orange. The KT positions (spheres) and trajectories (lines) of the Schromosome exhibiting independent movements are colored in red and purple. Note that one bivalent underwent hyperstretching, and then independent KT movement, followed by predivision of sister chromatids. Grid unit, 5 μm. Time after NEBD (hh:mm). See also Fig. 2a.

Supplementary Movie 5 Images of a young Sycp3-/- oocyte expressing 2mEGFP-CENP-C (green) and H2BmCherry (blue) were reconstructed into 3D. All homologous KTs were detected and tracked throughout MI. KT positions (spheres) and the connection of homologous KTs (lines). The colored spots indicate the KTs of univalents. The KT positions (spheres) and trajectories (lines) of univalents are colored in red and purple. The set of univalents underwent unbalanced predivision. Grid unit, 5 μm. Time after NEBD (hh:mm). See also Supplementary Fig. 4a.

Supplementary Movie 6 Images of an aged BDF1 oocyte at late metaphase I were reconstructed into 3D. The, first round of the movie shows the immunofluorescence signals for KTs (ACA, green) and DNA (Hoechst 33342, red). The second round shows the positions of sister KTs (white spheres and lines). The third round shows the connections between homologous KTs (red lines). The sister KTs of univalents were indicated by magenta spheres and lines. See also Fig. 3b.

Supplementary Movie 7 Images of an oocyte from a 28-year-old patient were reconstructed into 3D. The first round of the movie shows the immunofluorescence signals for KTs (ACA, green) and DNA (Hoechst 33342, red). The second round shows the positions of sister KTs (white spheres and lines). The third round shows the connections between homologous KTs (red lines). See also Fig. 4a.

Supplementary Movie 8 Images of an oocyte from a 42-year-old patient were reconstructed into 3D. The first round of the movie shows the immunofluorescence signals for KTs (ACA, green) and DNA (Hoechst 33342, red). The second round shows the positions of sister KTs (white spheres and lines). The third round shows the connections between homologous KTs (red lines). The sister KTs of univalents were indicated by magenta spheres and lines. See also Fig. 4b.
