# Supplementary material for: Bivalent separation into univalents precedes age-related meiosis I errors in oocytes
Source: Nat Commun. 2015 Jul 1;6:7550. doi: 10.1038/ncomms8550 (PMC4507009; doi:10.1038/ncomms8550)
Supplement: Supplementary Information — Supplementary Figures 1-6 and Supplementary Tables 1. [file ncomms8550-s1.pdf]

Supplementary Figure 1

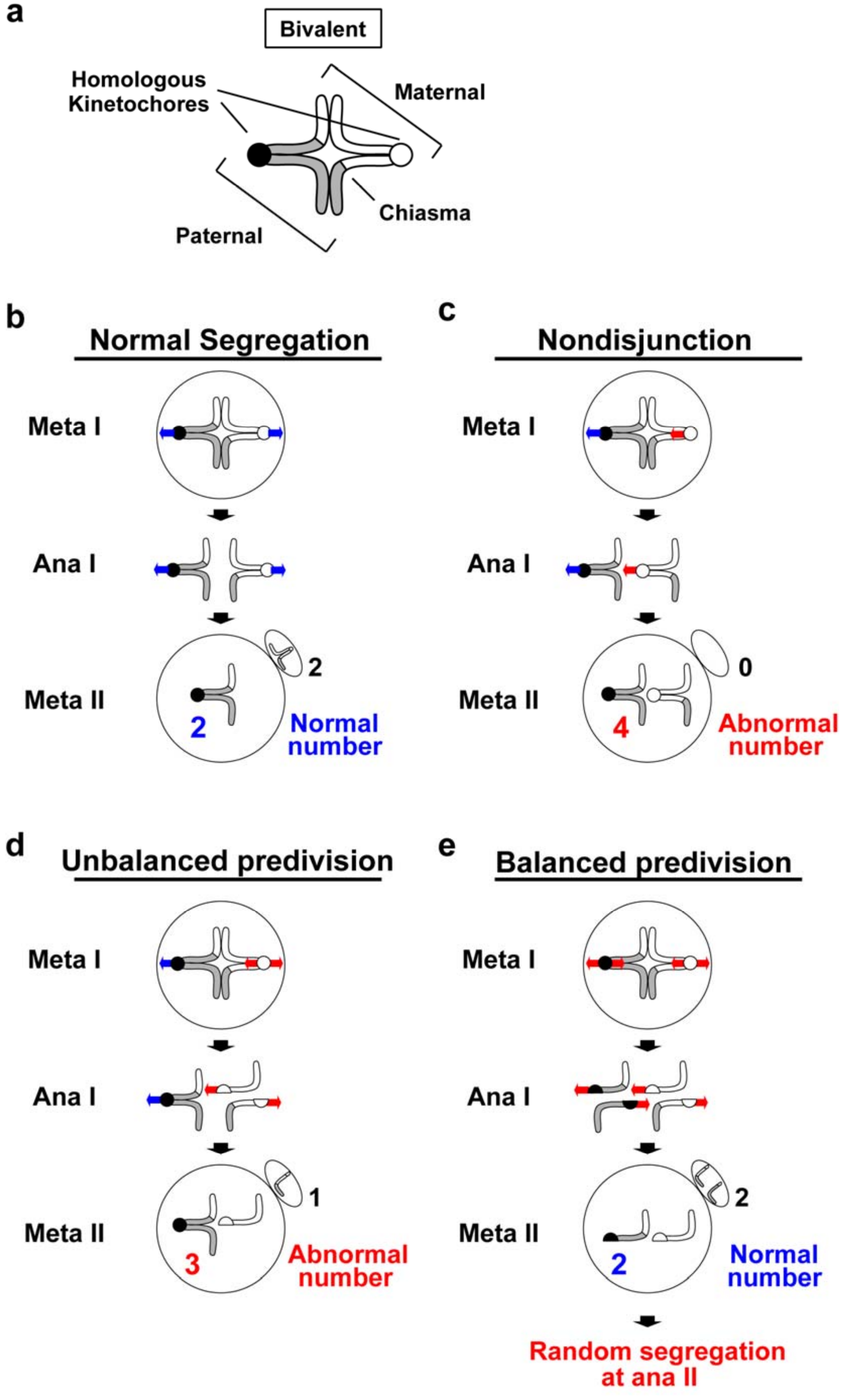

Supplementary Figure 1: Distinct types of chromosome segregation errors

(**a**) The bivalent structure. A bivalent is comprised of four chromatids and a pair of homologous KTs. Homologous chromosomes are physically connected by chiasmata.

(**b**) Normal chromosome segregation. One of the homologous KTs is pulled into one spindle pole, and the other is pulled into the opposite pole. This results in 2:2 segregation of chromatids.

(**c**) Nondisjunction of homologous chromosomes. One of the homologous KTs is pulled into one spindle pole, and the other is pulled into the same pole. This results in 4:0 segregation.

(**d**) Unbalanced predivision of sister chromatids. One of the homologous KTs is pulled into one spindle pole. The other is pulled into two spindle poles, which divides one pair of sister chromatids. This results in 3:1 segregation.

(**e**) Balanced predivision of sister chromatids. Both of the homologous KTs are pulled into two spindle poles, which divides both pairs of sister chromatids. This results in 2:2 segregation. Note that although this segregation results in the normal number of chromatids after MI, the centromere-proximal genetic pattern is abnormal because one of the chromatids carries a paternal centromere and the other carries a maternal centromere. Moreover, the chromatids are no longer paired after MI and thus undergo random segregation during MII.

Supplementary Figure 2

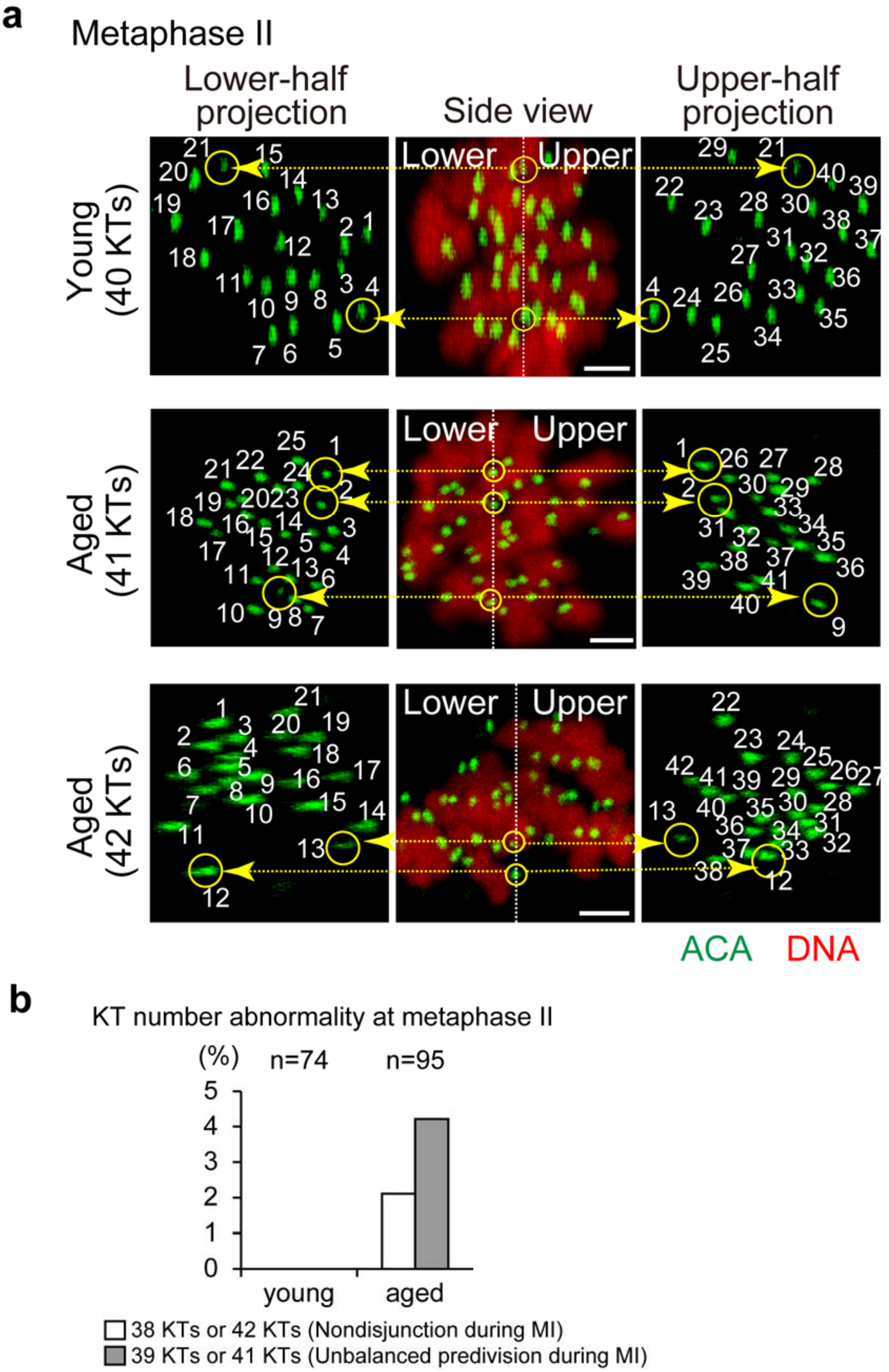

Supplementary Figure 2: Aneuploidy in oocytes at metaphase II

(**a**) KT number abnormality at metaphase II. Oocytes from young or aged BDF1 mice were fixed at metaphase II and immunostained for KTs (ACA, green). DNA was counterstained with Hoechst 33342 (red). Images were reconstructed into 3D with signal interpolation in z and the number of KTs was counted. Projection images of the half volume of oocytes are shown. Circles indicate identical KTs that are captured in both projection images. Scale bar, 2 μm. See also Supplementary Movie 3.

(**b**) KT number abnormalities were categorized into two groups. An odd number (39 or 41) of KTs results from unbalanced predivision during MI. An even number (38 or 42) of KTs results from nondisjunction during MI. Note that balanced predivision during MI results in the normal number of KTs (40) and thus is indistinguishable from premature sister chromatid separation during MII in this assay.

Supplementary Figure 3, a-f

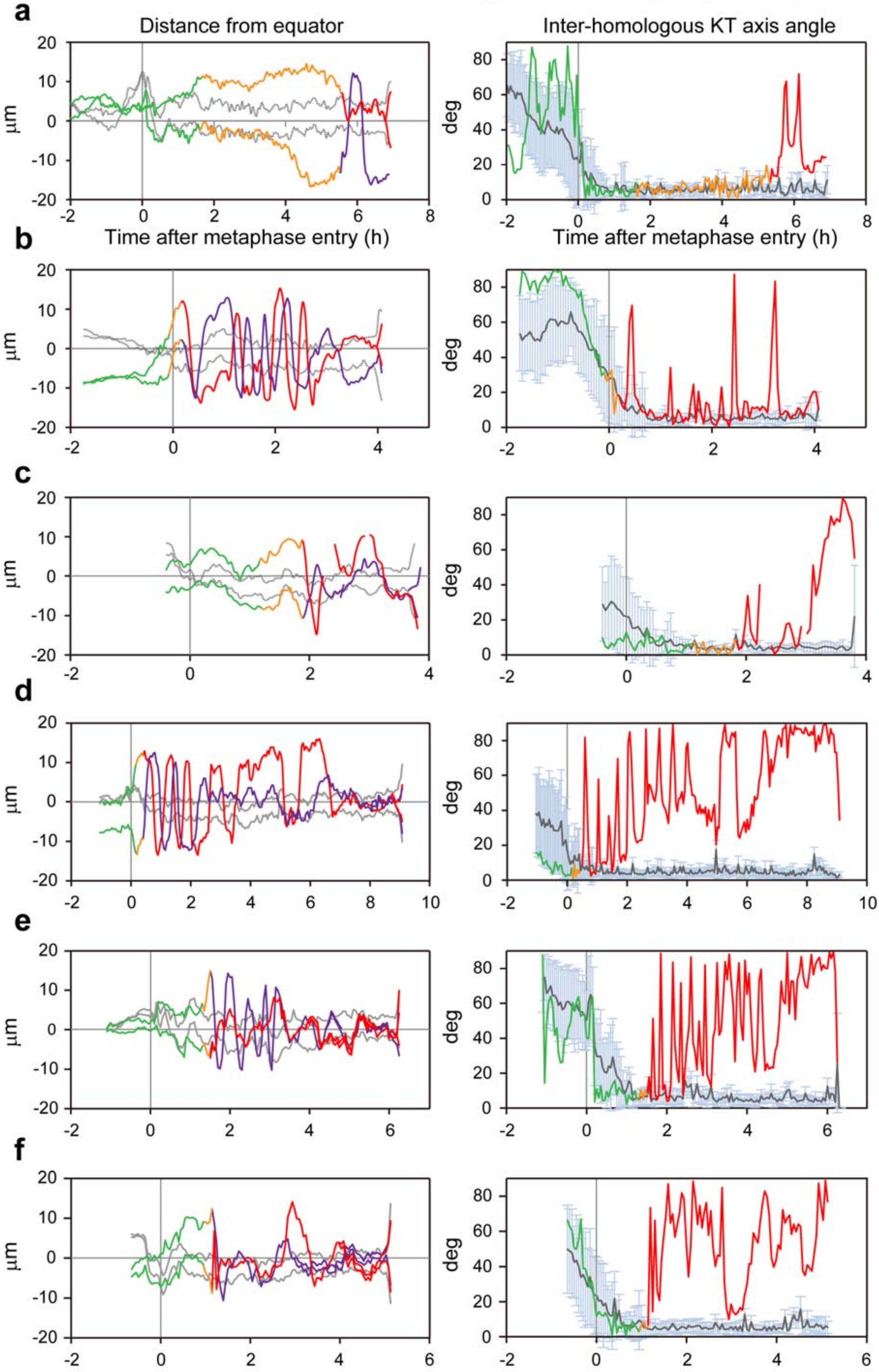

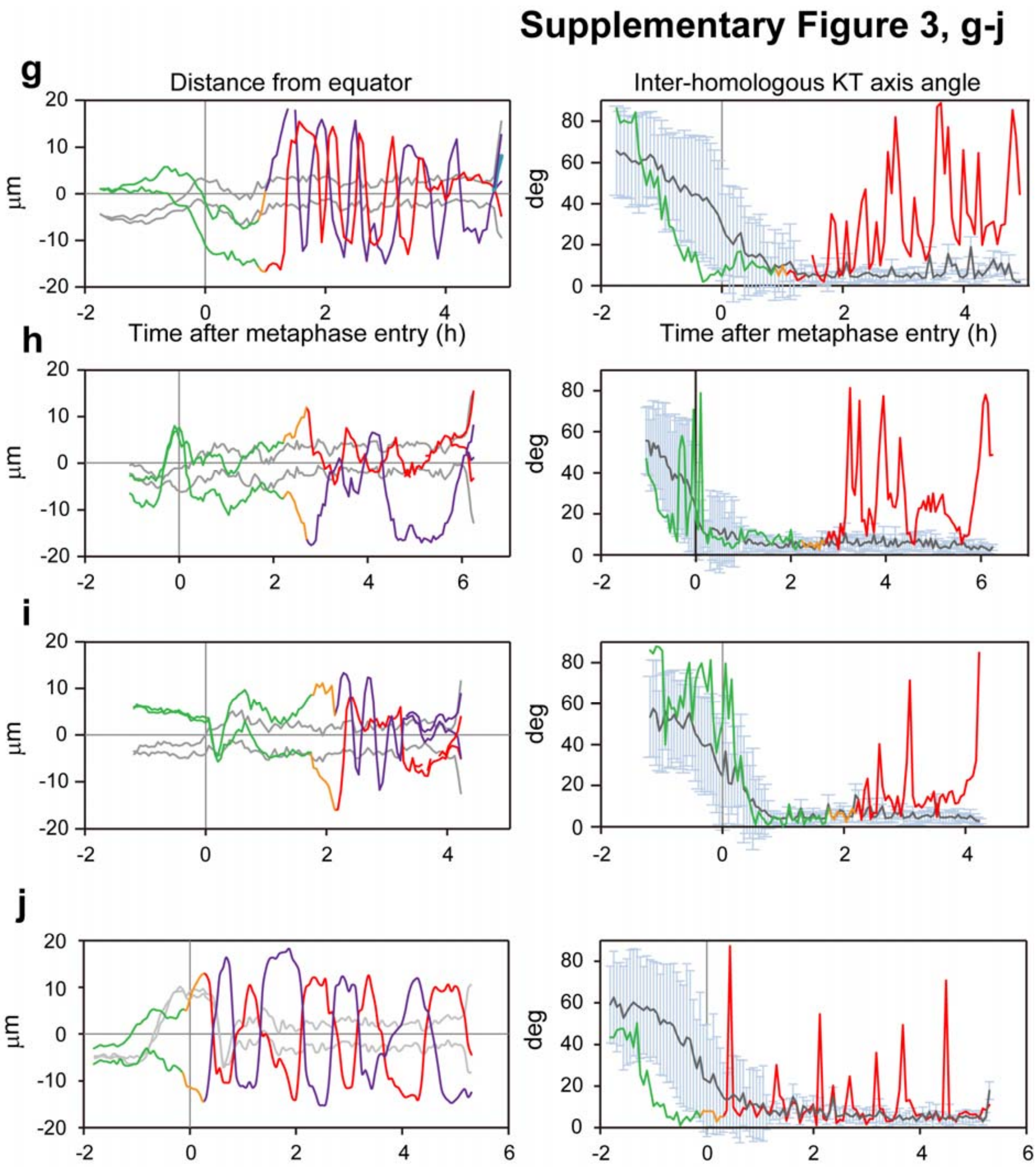

Supplementary Figure 3: Bivalent separation prior to segregation errors.

(**a–j**) KT trajectories prior to segregation errors suggest bivalent hyperstretching followed by separation into univalents. KT trajectories that resulted in unbalanced predivision (a–c), balanced predivision (d–i) and nondisjunction (j). Distance from the spindle equator (left) and angle between the inter-homologous KT axis and the spindle axis (right) are shown. The data are colored as follows: before hyperstretching (green), after hyperstretching (orange),

after initiation of independent KT movement (red and purple). The data for intact bivalents are colored in grey. Error bars, s.d.

Supplementary Figure 4

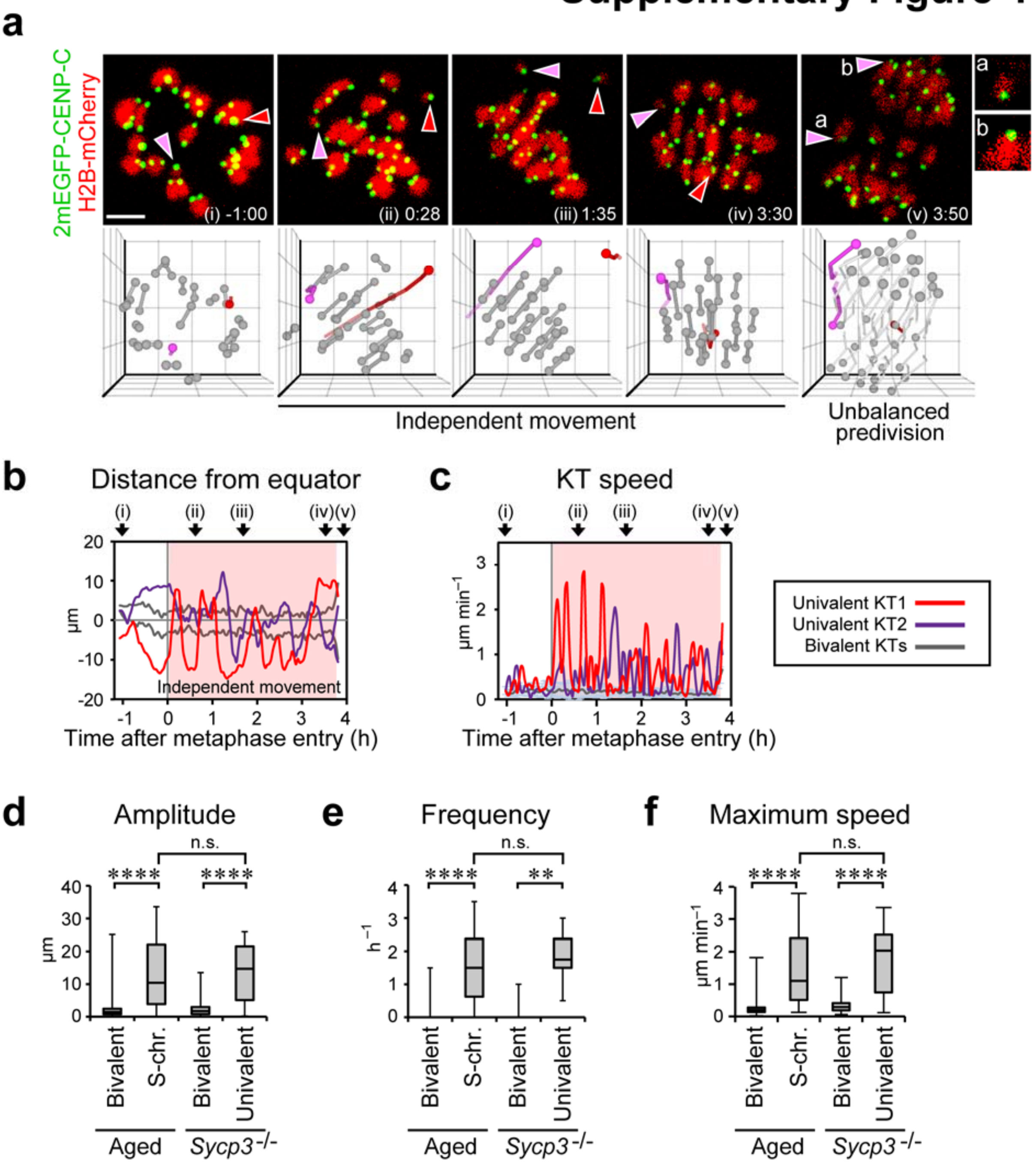

Supplementary Figure 4: Independent movement of S-chromosome KTs is indistinguishable from that of univalent KTs.

(**a**) Live imaging of univalents in young *Sycp3*$^{-/-}$ oocytes. Maximum z-projection images are shown. KT signals are peak-enhanced and background-subtracted. Arrowheads indicate univalent KTs. The 3D plot shows the KT positions (grey spheres) and the connection between homologous KTs (grey lines) of bivalents, and the KT positions (red and purple

spheres) and tracks (red and purple lines) of univalents. Unlike the oocytes of aged BDF1 mice, the oocytes of young *Sycp3*$^{-/-}$ mice contained univalents that were already separated before metaphase I (i). During early metaphase I, the univalents in the *Sycp3*$^{-/-}$ oocytes showed characteristic oscillations between the two spindle poles (ii–iii). The set of the univalents underwent unbalanced sister chromatid predivision (v, arrowheads and insets). Time after metaphase entry (h:mm). Scale bar, 5 μm. See also Supplementary Movie 5.

(**b–c**) Univalent dynamics in young *Sycp3*$^{-/-}$ oocytes. KT distance from the spindle equator (b) and KT speed (c). The data for univalent KTs are colored as in (a). The data for bivalents are colored in grey. Note that the univalents exhibit independent oscillation with an increased amplitude, frequency, and speed compared with those of bivalents after metaphase entry. Error bars, s.d.

(**d-f**) The characteristic profiles of univalent oscillation in young *Sycp3*$^{-/-}$ oocytes are similar to those of independent movement of S-chromosome KTs in aged BDF1 oocytes. S-chromosome dynamics for 2 hours after the initiation of independent KT movements in aged BDF1 oocytes and univalent dynamics for 2 hours after metaphase entry in young *Sycp3*$^{-/-}$ oocytes were analyzed. Oscillation amplitude (n=2416, 135, 382, 37) (d), frequency (n=418, 22, 114, 6) (e) and maximum KT speed during oscillation (n=2416, 135, 382, 37) (f). Boxes show the 25th to 75th percentiles and whiskers show the minimum to maximum. Two-tailed, unpaired Student's *t*-test was performed. **p<0.01; ****p<0.0001.; n.s., not significant.

Supplementary Figure 5

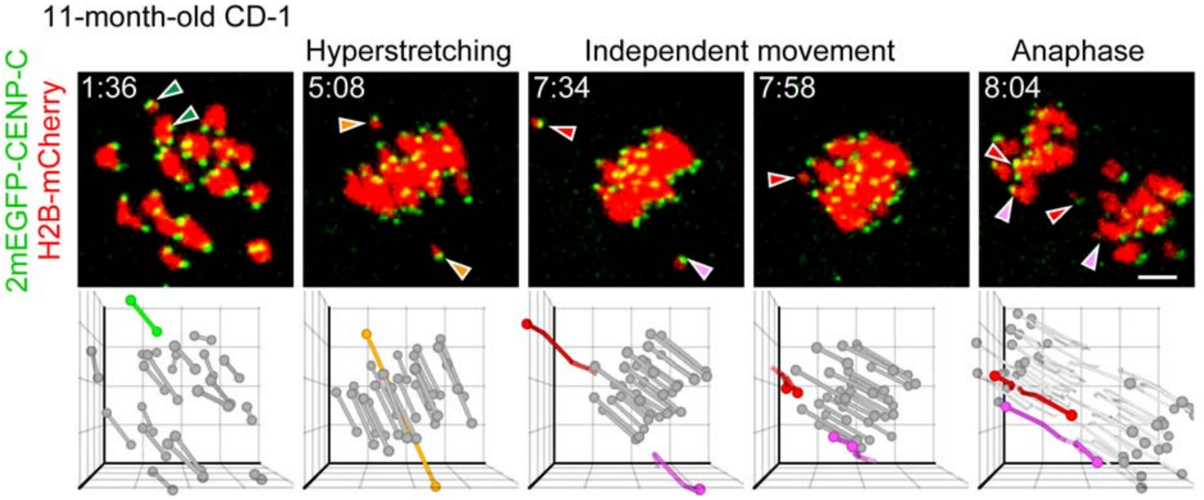

Supplementary Figure 5: Premature bivalent separation prior to segregation errors in the oocytes of aged CD-1 mice

Live imaging of the oocytes of 11-month-old CD-1 mice. Maximum z-projection images are shown. KT signals are peak-enhanced and background-subtracted. Green arrowheads indicate the KTs of a bivalent before hyperstretching. Orange arrowheads indicate the KTs of the hyperstretched bivalent. Red and purple arrowheads indicate separated chromosome units exhibiting independent movement. They underwent balanced predivision of sister chromatids at anaphase. The 3D plots show KT positions (spheres) and the connection between homologous KTs (lines) or KT trajectories (lines) after the initiation of independent movement. Spheres and lines were colored as in arrowheads. Time after NEBD (h:mm). Scale bar, 5 μm.

Supplementary Figure 6

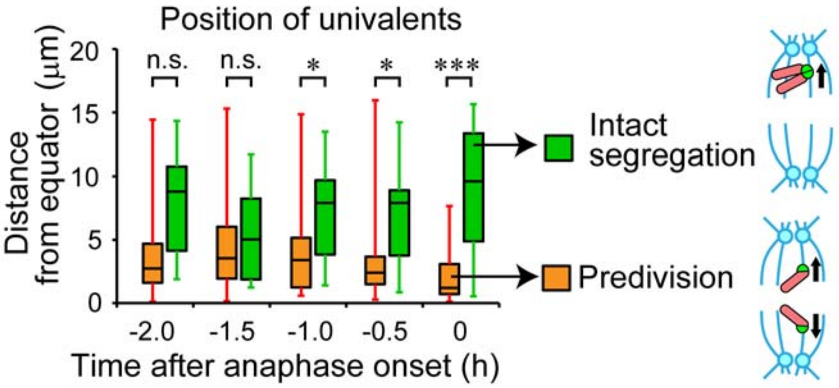

Supplementary Figure 6: Univalents localize near the spindle equator prior to predivision

The univalents in live aged BDF1 oocytes were categorized into those that underwent intact segregation and those that underwent predivision. The distance of univalents from the spindle equator prior to anaphase I is shown (n = 15, 17). Two-tailed, unpaired Student's *t*-test was performed. *$p<0.05$, ***$p<0.001$.

Supplementary Table 1

Mouse	NEBD	Bivalent state during MI (%)		Anaphase I (%)	Type of segregation during MI (%)	
Young BDF1 (2-mo)	211	Bivalent	209 (99.1)	167 (79.9)	Normal	167 (100)
		Hyperstretching → Univalents	2 (0.9)	0 (0)		
Aged BDF1 (16-mo)	383	Bivalent	331 (85.0)	243 (73.4)	Normal	238 (98.0)
					Nondisjunction	2 (0.8)
					Unbalanced predivision	1 (0.4)
		Hyperstretching	13 (3.3)	11 (84.6)	Normal	10 (90.9)
					Unbalanced predivision	1 (9.1)
		Hyperstretching → Univalents	37 (9.5)	19 (51.4)	Normal	5 (26.3)
					Unbalanced predivision	4 (21.1)
					Balanced predivision	9 (47.4)
					Nondisjunction	1 (5.3)
		Two hyperstretching → Two sets of univalents	2 (0.5)	2 (100)	Unbalanced predivision + Nondisjunction	1 (50)
					ND*	1 (50)
Aged CD-1 (11-mo)	151	Bivalent	133 (88.0)	126 (94)	Normal	124 (98.0)
					Nondisjunction	2 (1.5)
					Unbalanced predivision	0 (0)
		Hyperstretching	2 (1.3)	2 (100)	Normal	2 (100)
					Unbalanced predivision	0 (0)
		Hyperstretching → Univalents	16 (10)	13 (81)	Normal	2 (15.4)
					Unbalanced predivision	3 (23.1)
					Balanced predivision	7 (53.9)
					Nondisjunction	1 (7.7)

* Not determined, because of failure to track four univalents. Two of the four univalents underwent predivision.

Supplementary Table 1: Summary of live imaging results.

Oocytes from young BDF1 (2-month-old), aged BDF1 (16-month-old), and aged CD-1 (11-month-old) mice were categorized based on live imaging results. We found two aged BDF1 oocytes that had a chromosome lacking its KT, which were not included in this table.
